# PTSD is associated with impaired event processing and memory for everyday events

**DOI:** 10.1186/s41235-022-00386-6

**Published:** 2022-04-25

**Authors:** Barbara L. Pitts, Michelle L. Eisenberg, Heather R. Bailey, Jeffrey M. Zacks

**Affiliations:** 1 Department of Psychological Sciences, Kansas State University, 942 Bluemont Hall, 1114 Mid-Campus Dr. North, Manhattan, KS 66503 USA; 2 Washington University in St. Louis, St. Louis, MO USA

**Keywords:** PTSD, Symptom severity, Event segmentation, Narrative priming, Memory

## Abstract

Current theories of posttraumatic stress disorder (PTSD) propose that memory abnormalities are central to the development and persistence of symptoms. While the most notable memory disturbances in PTSD involve memory for the trauma itself, individuals often have trouble remembering aspects of everyday life. Further, people with PTSD may have difficulty segmenting ongoing activity into discrete units, which is important for our perception and later memory of the activity. The current study investigated whether PTSD diagnosis and symptom severity predicted event segmentation and memory for everyday activities. To do so, 63 people with PTSD and 64 controls with a trauma history watched, segmented, and recalled videos of everyday activities. Viewers with higher PTSD symptom severity showed lower agreement on locations of event boundaries and recalled fewer fine-grained actions than did those with lower symptom severity. These results suggest that PTSD symptoms alter event segmentation, which may contribute to subsequent memory disturbances.

**Supplementary Information:**

The online version contains supplementary material available at 10.1186/s41235-022-00386-6.

Post-traumatic stress disorder (PTSD) is a psychiatric disorder experienced at clinical levels by roughly 6.8% of US adults (Kessler et al., 2005). Symptoms of PTSD include avoidance of trauma reminders, negative thoughts and mood, and alterations in arousal and reactivity (American Psychiatric Association, 2013). Many current theories of PTSD agree that memory abnormalities are central to the development and persistence of symptoms (Brewin, 2011; Rubin et al., 2008). The most notable memory disturbances in PTSD involve memory for the trauma itself and are characterized by both vivid involuntary flashbacks of the event and fragmented, disorganized voluntary memories of the event (Brewin, 2011), but there is some debate as to the relative importance of objective (Jones et al., 2007) versus subjective or metacognitive (Bennett & Wells, 2010) memory problems driving symptoms of PTSD.

In addition to disturbances in trauma-related memory, individuals with PTSD often self-report trouble remembering aspects of everyday life. For example, combat veterans with PTSD report greater frequency and seriousness of forgetting, more change in memory ability, and less mnemonic usage than non-combat controls (Carlozzi et al., 2011). These subjective memory complaints are consistent with evidence that PTSD symptom severity predicts objective memory deficits on neuropsychological tests (Scott et al., 2015). These deficits in everyday memory negatively affect social and occupational functioning (Geuze et al., 2009) and treatment outcomes (Wild & Gur, 2008). Therefore, understanding PTSD’s effect on everyday memory function may help develop more specific treatments and improve functional outcomes (Scott et al., 2015).

This chronic impairment to everyday memory may be further exacerbated by periods of heightened symptoms, such as when a flashback is experienced. Reminders of a traumatic event have been shown to provoke physiological arousal (Pitman et al., 1987) and to activate networks associated with brain abnormalities in individuals with PTSD (Britton et al., 2005). For example, Britton et al. (2005) examined response to trauma reminders in combat veterans with PTSD, combat veterans without PTSD, and healthy controls. In this study, a narrative script of the participant’s own traumatic experience elicited significantly greater ratings of negative emotions and fear and a greater psychophysiological response, than a neutral script for both PTSD patients and control participants who had experienced combat trauma. Further, the traumatic reminders altered activity in brain areas related to both cognitive processing and PTSD dysfunction, such as the amygdala, medial frontal cortex, and the anterior cingulate cortex in PTSD patients relative to both control groups, suggesting that these brain regions may not function properly after a trauma reminder (Britton et al., 2005). However, the effect of heightened emotional arousal from a trauma reminder on processing of everyday stimuli is largely untested.

Sherrill and Magliano (2017) suggested that theories of event perception could help to explain everyday cognitive functioning deficits associated with PTSD. As they point out, theories of PTSD argue that symptoms are causally related to maladaptive encoding, storage, and retrieval of trauma memories (Brewin et al., 2010). In contrast to traditional memory measures that cannot distinguish between these memory processes, event perception paradigms offer an opportunity to examine encoding processes in real time, while an individual is perceiving an event. For example, Eisenberg et al. (2016) observed that PTSD symptom severity was associated with worse event processing, and they hypothesized that this effect is due to hypervigilance that distracts people with PTSD from relevant stimuli and draws their attention towards irrelevant perceptual information. In line with this proposed explanation, Sherrill et al. (2019) found that anxiety from viewing a stressful film led to more perceptual and less conceptual encoding. This finding is also consistent with cognitive models of PTSD, which suggest that encoding is affected by an attentional bias toward perceptual information and away from conceptual information (Ehlers & Clark, 2000). Eisenberg et al. (2016) hypothesized that encoding deficits associated with PTSD may affect the ability to orient to what is most relevant, make and monitor predictions about what will occur next, and update memory at appropriate intervals. For example, while driving, people with PTSD may be distracted looking for potential threats along the roadside and miss important cues that something is going to happen on the road in front of them. Those missed cues will likely cause them to form different event models and subsequent memories of their drive. These hypotheses can be tested using event perception theories.

To make sense of the vast amount of perceptual information in the environment, people mentally break up or “chunk” activity into discrete events. Event Segmentation Theory (Zacks et al., 2007) offers a theoretical framework for how this perceptual process operates. According to Event Segmentation Theory, perceptual processing is driven by both incoming sensory information and prior relevant experiences, both of which are mentally represented in an event model in working memory. The perceptual system uses the event model to predict what will occur in the near future. When activity becomes less predictable, the event model is updated to better reflect what is happening. This period of model updating is experienced by the perceiver as an *event boundary*.

To evaluate the perception of event boundaries, a segmentation (or “unitization”) task is often used (Newtson, 1973). This task requires that participants view a video and press a button when, in their opinion, one meaningful activity has ended and a new one has begun. For example, an observer watching a video of an actor doing dishes is likely to press a button after each cleaned dish is placed on the towel to dry. Boundary perception coincides with both perceptual (Zacks et al., 2009) and conceptual (Zacks et al., 2010) changes in ongoing activity. In the dishwashing example, the point in time when the actor sets a plate on the towel involves both a relative increase in perceptual change, such as the actor turning their body towards the towel and the plate moving across the screen from the sink to the towel, and a point of conceptual change, as the actor has completed the subgoal of washing the plate and is ready to move on to a new subgoal.

Participants tend to largely agree on where they perceive such event boundaries (Zacks et al., 2007). S*egmentation agreement* is a measure of how well a participant’s event boundary locations agree with those of the group. Higher segmentation agreement amounts to selecting event boundaries that are normative. Importantly, segmentation agreement strongly predicts subsequent memory, and does so above and beyond other cognitive abilities (Sargent et al., 2013). That is, participants with lower segmentation agreement (i.e., less able to identify event boundaries) are also more likely to recall fewer actions from the event (Bailey et al., 2013; Flores et al., 2017; Newberry & Bailey, 2019; Pitts et al., 2022).

Event boundaries serve as important anchors in memory. For example, people remember more actions (Schwan et al., 2000) and scenes (Huff et al., 2014) from boundaries than non-boundaries. Removing intervals that contain event boundaries impairs memory more than removing intervals from event middles (Schwan & Garsoffky, 2004), while making boundaries more salient using cues improves memory (Gold et al., 2017). Furthermore, memory for recent information decreases immediately after a boundary (Swallow et al., 2009). Thus, event boundaries help to organize activity in long-term memory by highlighting points of interest in action and binding together the information within an event (DuBrow & Davachi, 2013).

In fact, Sargent et al. (2013) suggested that event segmentation ability may mediate the effect of age on memory performance in older adults. Older adults show both reduced segmentation ability and poorer episodic memory compared to young adults, and importantly, segmentation agreement is related to memory performance, particularly in older adults (Kurby & Zacks, 2011). Taken together, these results suggest that older adults have difficulty identifying meaningful structure of everyday events, and that this difficulty contributes to memory deficits. Despite these suggestions, previous research has not explicitly investigated the mediating role between age and memory performance.

Very little work has investigated event perception in PTSD, but there is some evidence of an event segmentation deficit. As described above, Eisenberg et al. (2016) found that PTSD symptom severity was associated with segmentation and memory performance in a non-clinical sample: Participants with greater symptoms segmented videos of everyday events less normatively, and also recalled less information about the activities. Furthermore, segmentation agreement was correlated with event memory. This study provided initial evidence that PTSD symptoms in a non-clinical, community sample, are associated with difficulty segmenting ongoing activity into meaningful units, which is, in turn, related to everyday memory function. Similar to suggestions from the aging literature, these results suggest that event segmentation may mediate the relationship between PTSD and memory deficits and help to explain the reason for these deficits.

A better understanding of the specific cognitive deficits associated with PTSD would help to better characterize the everyday functional impairments experienced by people with this disorder. While most research focuses on memory for the traumatic event, the research reviewed here suggests a more widespread event perception and memory deficit that has a broader impact on daily life. In fact, everyday memory difficulties may be a more debilitating issue than our current conceptualization of PTSD suggests.

The current study investigated event segmentation and memory for everyday activities in patients diagnosed with PTSD and people with a trauma history but not diagnosed with PTSD, who were matched on age, gender, years of education, and ethnicity. Participants watched, segmented, and remembered several videos of everyday activities. Additionally, they listened to narratives of their traumatic event prior to some videos, so that we could test the effect of heightened arousal on event cognition and memory. An important limitation of previous research is that it examined PTSD symptoms within a community sample that included few people who might be diagnosed with PTSD (Eisenberg et al., 2016). In this study, we recruited matched participants with and without a PTSD diagnosis, all of whom had experienced significant trauma. Because diagnostic categorization of PTSD is not perfectly reliable (Foa et al., 2016; Weathers et al., 1993) and because prior work has shown that symptom severity is associated with cognitive performance (e.g., Eisenberg et al., 2016; Scott et al., 2015), we did not rely solely on the dichotomous classification of participants; instead, before investigating any a priori hypotheses, we examined the variability in PTSD severity among the control and PTSD groups. Because there was a high level of variability in PTSD severity, especially in the PTSD group, we assessed the relationship between PTSD pathology and event processing using two types of model: models that measured effects of PTSD diagnosis, and models that measured levels of PTSD symptom severity as a continuous variable.

We hypothesized that people with PTSD and people with higher symptom levels would show worse event segmentation ability and long-term memory than controls and those with lower symptom levels. Further, we hypothesized that these PTSD-related deficits in segmentation agreement and memory would be exacerbated after being primed with their trauma narrative relative to their positive narrative. Finally, we hypothesized that segmentation agreement would mediate the PTSD-related deficit in everyday memory performance, such that people with PTSD and those with higher symptom levels would show worse event segmentation and would, in turn, show lower memory performance. Such findings would suggest that the relationship between PTSD symptoms and memory for everyday events (Eisenberg et al., 2016) is clinically relevant and therefore has implications for our understanding of and the treatment of PTSD.

## Method

### Participants

Participants were 18- to 50-year-olds with a history of a traumatic life experience, recruited from the Volunteer for Health participant registry, a subject pool maintained by the Washington University School of Medicine and from advertisements posted on Saint Louis Craigslist. All potential participants were screened in a phone interview using the Mini Neuropsychiatric Interview (Sheehan et al., 1998) to determine eligibility and preliminary assignment to groups. Potentially eligible participants (n = 194) were screened at the first study session using the SCID to verify eligibility, confirm PTSD diagnosis, and determine final group assignment. Control participants were recruited to match PTSD participants on age (within 10 years), gender, years of education (within approximately two years), and ethnicity (if mixed ethnicity, at least one match) and were required to have experienced a traumatic event that met the A1 DSM-IV criterion for PTSD.

Exclusion criteria for the PTSD group included no PTSD diagnosis at session 1, history of psychosis, current substance use disorder, and current manic episode. Exclusion criteria for the control group included more than three current PTSD symptoms, PTSD symptoms that significantly interfered with important life functioning or that caused significant distress, history of psychosis, current substance use disorder, and current manic episode.

Table 1 provides demographic information on the final sample.

**Table 1:** Demographic and diagnostic characteristics of sample

	PTSD	Control	*p*-value
Final sample	63	64	
Mean age in years (SD)	34.60 (8.82)	34.11 (9.68)	.764
Mean years of education (SD)	14.37 (1.80)	14.80 (1.79)	.186
Gender			
Male	12	11	
Female	51	53	
Racial identification			
White	34	44	
Black	14	16	
Asian	2	1	
Mixed race	12	3	
Unknown	1	0	
Mean (SD) psychological scores			
PCL	54.60 (13.66)	20.81 (6.21)	< .001
DASS	57.21 (28.95)	9.70 (14.74)	< .001
DES	670.56 (478.52)	182.19 (211.96)	< .001
LSAS	64.93 (28.35)	30.36 (21.64)	< .001
MPSS	52.78 (18.04)	66.38 (15.26)	< .001

Note: *PCL *PTSD Checklist, *DASS* Depression Anxiety Stress Scale, *DES *Dissociative Experiences Scale, *LSAS* Liebowitz Social Anxiety Scale, *MPSS* Multidimensional Scale of Social Support

## Materials and measures

### Videos

Six videos were shot at a rate of 25 fps and depicted actors (college students) performing activities typical in everyday life, including making breakfast (329 s), preparing for a party (376 s), planting plants (354 s), walking through a library (249 s), sweeping (263 s), and doing dishes (327 s). All videos were filmed from a fixed, head-height perspective, with no pan or zoom. Initial video presentation was randomized and order was counterbalanced across participants.

### Psychological assessments

Clinical diagnoses, including a diagnosis of PTSD, were assessed using the Structured Clinical Interview for DSM-IV-Research Version (SCID-IV; Mood, Substance Use, PTSD, and Psychosis Modules; First et al., 2002). The SCID is one of the most widely used diagnostic instruments in clinical research and has high clinical validity and reliability (First & Gibbon, 2004). The SCID was modified to include criteria for both DSM-IV and DSM-5, as the SCID for DSM-5 had not yet been released at the time the study began.

The PTSD Checklist for DSM-IV—Civilian Version (PCL-C; Weathers et al., 1993) measured self-reported symptoms of PTSD identified by the DSM-IV-TR (American Psychiatric Association, 2000). Respondents rated on a 5-point scale ranging from 1 (*not at all*) to 5 (*extremely*) how much “you have been bothered by that problem in the past month.” The 17 items were summed for a total score with a possible range of 17–85. This brief screening tool is one of the most well accepted of PTSD symptoms. The updated version of this measure (PCL-5; Weathers et al., 1993) was not available when data collection first began in 2013.

Additionally, participants completed the Depression Anxiety Stress Scale-42 (DASS; Lovibond & Lovibond, 1995), the Dissociative Experience Scale (DES; Bernstein & Putnam, 1986), the Liebowitz Social Anxiety Scale (Liebowitz, 1987), and the Multidimensional Scale of Perceived Social Support (MPSS; Zimet et al., 1988).

### Free recall task

Immediately after watching each movie, participants were given seven minutes to type as much as they could remember from the movie they had just watched, in the order they remembered the activity occurring. To score the free recall, research team members constructed a list of the basic actions performed by the actor in the video using the action coding system (ACS) described by Schwartz et al. (1991). The ACS constructs a hierarchy of action sequences, which consist of low-level A1 units grouped into higher-level A2 units. A1 units are the basic actions involved in completing a higher-level goal (i.e., picking up a plate, scrubbing the plate, rinsing the soap off, and setting the plate on a towel). A2 units are one step higher than an A1 and encompass many A1s to satisfy a higher-level sub-goal (i.e., wash a plate). After familiarization with the scoring procedure, each research team member scored ten participant responses for one video, and item scores were compared with those of one of the lead researchers. This initial scoring produced an interrater Kappa = 0.90. Discrepancies were reviewed and discussed to agree on general scoring principles. Each team member then coded the remaining participant recall responses for one video. The proportion of correctly recalled A1 units (i.e., number of A1 units recalled/ total number of A1 units in that video) for each video was the dependent measure. Of note, responses to this task generally do not contain inaccurate information or intrusions.

### Event segmentation task

While watching each video, participants were instructed to press a button whenever they believed a meaningful unit of activity had ended and another had begun. Previous studies using this method have observed a high degree of similarity in the locations at which people button press during this task (Zacks et al., 2007). A segmentation agreement score was calculated for each participant. Segmentation agreement is a measure of similarity between each participant’s segmentation behavior and the segmentation of a reference group, which in this study is the whole sample. This measure was calculated using the methods described in Kurby and Zacks (2011). Time in each of the movies was divided into one-second bins. Then, sample norms for segmentation were calculated by determining the proportion of participants who identified an event boundary within each one-second bin. Segmentation agreement scores were generated by correlating each participant’s segmentation with the sample segmentation norm. The resulting correlations were rescaled to between 0 and 1 to control for individual differences in the number of boundaries identified. This rescaling (see Kurby & Zacks, 2011) helped to correct for the restricted range of correlations from participants who chose either an exceptionally low or high number of boundaries.

### Life event narratives

Participants wrote brief narratives of one traumatic event and one positive event from their own lives. Participants listened to recordings of their narratives before each task during the second and third sessions. Half of the participants heard the traumatic narrative in the second session and the positive narrative in the third session; and the other half heard the positive narrative in the second session and the traumatic narrative in the third session. After listening to each recording, participants rated their mood and anxiety on a 10-point Likert scale. This procedure (recording then measurement) occurred 11 times over the course of each session and each time was recorded as a separate trial. This narrative priming approach has been found to increase physiological arousal during recollection of traumatic experiences (Pitman et al., 1987).

### Eye tracking

Gaze location from the participants’ right eye was tracked using an eye tracker (EyeLink 1000; SR Research Ltd, Mississauga, ON, Canada) that sampled at 1000 Hz. Results from this measure are reported in Eisenberg et al. (in prep), which focuses on the relationship between PTSD and prediction ability. These data will not be addressed in the current study because they do not directly interrogate the relationship between PTSD, event segmentation ability, and memory that is hypothesized here.

### Explicit prediction task

Participants watched three movies of an actor performing an everyday activity. The movies were paused at eight times during each movie, and participants were asked to choose which of two movie frames they believed would occur five seconds later in the movie. Further information on the design and results from this task is reported in Eisenberg, Rodebaugh, Flores, and Zacks (in prep).

### Procedure

Participants completed tasks across three sessions on three different days. During the first session, participants completed informed consent, psychological assessments, and wrote a description of a positive and a traumatic life event narrative to be used in priming autobiographical memories in the subsequent sessions. The first session was 2–4 h long. During the second session, which occurred on average 9.8 days (median = 7 days; range = 1–146 days) after their assessment session, participants listened to an audio recording (read by a member of the research team) of either the traumatic or positive autobiographical event narrative they wrote in session 1 and completed four event comprehension tasks: (1) an explicit prediction task; (2) eye-tracking during passive viewing of videos; (3) a free recall test on each of the videos; and (4) an event segmentation task on each of the videos. The order of the explicit prediction task and the eye-tracking/ recall/ event segmentation tasks were counterbalanced across participants, such that participants either completed the prediction task first or completed the eye-tracking/ recall/ and event segmentation tasks first. During the third session, which occurred on average 10.2 days after the second session (median = 7 days; range = 3–77 days), participants completed the same tasks, but after being reminded of the other autobiographical event they recorded in session 1 and with novel videos in each task. The order of autobiographical event (traumatic vs. positive) was counterbalanced across participants. Participants were given a chance to ask questions about the study at the end of the third session. The second and third sessions were each two hours long.

## Results

The analyses reported here follow the reasoning laid out in the Introduction. First, we conducted a manipulation check, testing that priming trauma narratives would increase anxiety; we also tested the hypothesis that priming of anxiety would be stronger in people with PTSD. Second, we tested the hypothesis that people with PTSD have poorer event segmentation ability than do controls and that this difference is exacerbated after being primed with their trauma narrative relative to their positive narrative. Third, we tested the hypothesis that people with PTSD would recall less information than controls and that this difference is exacerbated after being primed with their trauma narrative relative to their positive narrative. Finally, we tested the hypothesis that segmentation agreement mediates the relationship between PTSD and memory performance. For each of these hypotheses, we tested two models: one to test the effect of the diagnosis of PTSD and a second to test the effect of PTSD symptom severity.

### Narrative priming

#### Group analysis

To determine whether the Narrative Priming task affected state anxiety, a mixed-effects model examined the fixed effect of Group (PTSD vs. Control), Priming Condition (Traumatic vs. Positive), and their interaction on average Anxiety ratings for each session. Subject and trial were treated as random effects. There was a significant effect of Group, such that those with PTSD (*M* = 3.71) reported higher overall Anxiety than those in the Control group (*M* = 2.06), *B* = 1.05, *p* < 0.001. There was also a significant effect of Priming Condition, such that Anxiety ratings were higher after the traumatic narrative (*M* = 3.57) than the positive narrative (*M* = 2.19), *B* = 0.83, *p* < 0.001. The Group x Priming Condition interaction was also significant, *B* = 1.01, *p* < 0.001. While both groups reported higher Anxiety after the Traumatic narrative compared to the Positive narrative, the PTSD group showed a larger difference between priming conditions than the Control group.

#### Symptom severity analysis

We then examined the effect of PTSD Symptom Severity (mean-centered), Priming Condition, and their interaction on average Anxiety ratings. Subject and trial were treated as random effects. There was a significant effect of Symptom Severity, B = 0.03, *p* < 0.001, Priming Condition, B = 1.35, *p* < 0.001, and their interaction, B = 0.04, *p* < 0.001. The relationship between symptom severity and anxiety––i.e., those with more severe symptoms reported more anxiety––was stronger after the traumatic priming than the positive priming (see Additional file 1: Figure S1).

### Event segmentation

**Fig. 1:**
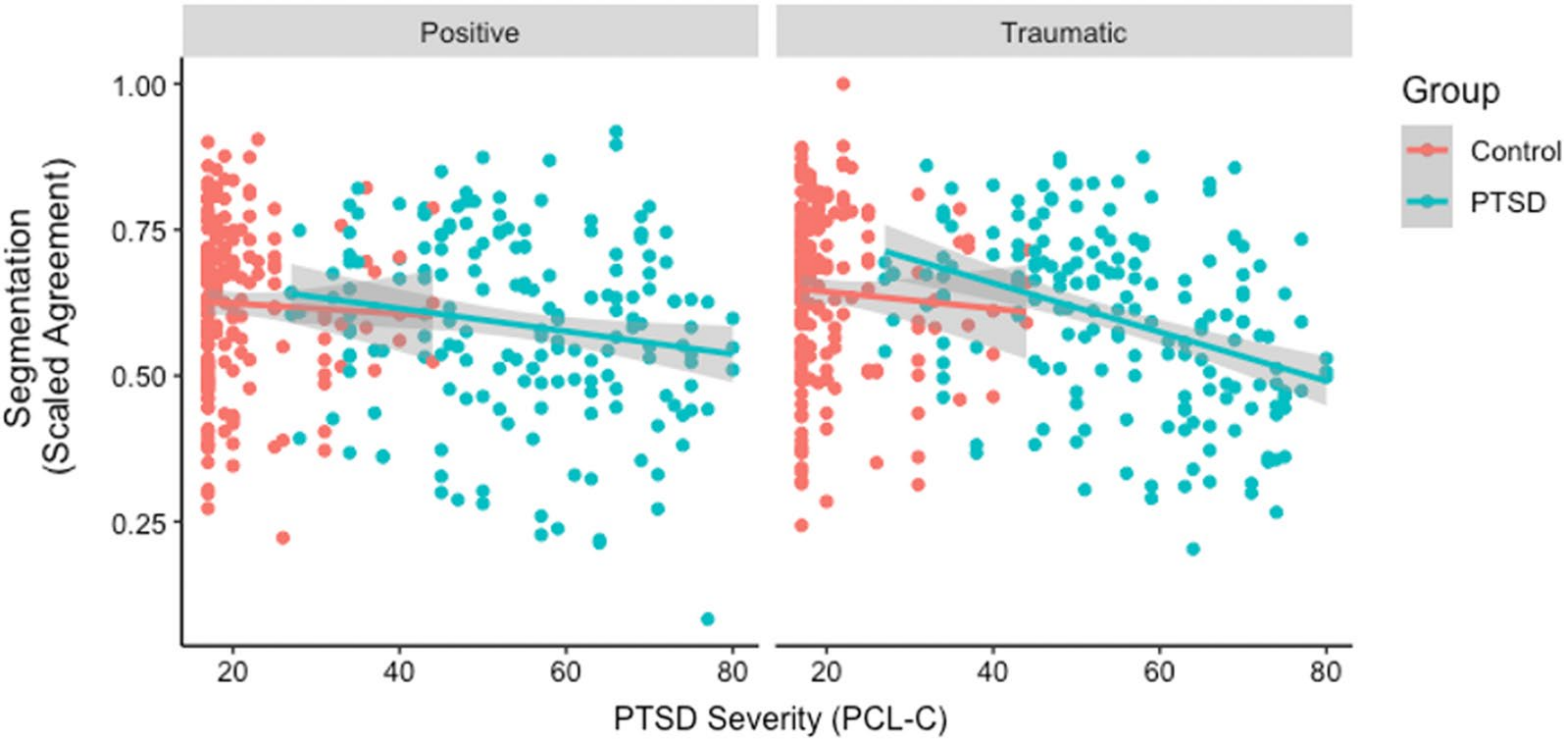
Higher PTSD symptom severity was associated with lower segmentation agreement, especially after the traumatic narrative. Blue dots = PTSD Group; Red dots = Control Group

Overall, participants identified an average of 28.68 (*Range* = 1–142, SD = 19.67) boundaries per video. Figure 1 plots the relationship between PTSD Symptom Severity and segmentation agreement by group.

#### Group analysis

To evaluate the effects of PTSD and narrative priming on segmentation, we ran a mixed-effects model predicting the fixed effects of Group (PTSD vs. Control), Narrative Type (Traumatic vs. Positive), and their interaction on segmentation agreement. The Subject and Video were treated as random effects. There was not a significant effect of Group (PTSD *M* = 0.59; Controls *M* = 0.63), *B* = −0.04, *p* = 0.056, nor Narrative Type (Positive narratives *M* = 0.60; Traumatic narratives *M* = 0.62), *B* = 0.02, *p* = 0.095, nor a significant interaction of Group × Narrative type, *B* = −0.01, *p* = 0.492.

#### Symptom severity analysis

To evaluate the effects of PTSD symptom severity and narrative priming on segmentation, we ran a mixed-effects model predicting the fixed effects of Symptom Severity (mean-centered), Narrative Type (Traumatic vs. Positive), and their interaction on Segmentation agreement. Subject and Video were treated as random effects. In this model, the fixed effect of Symptom Severity was significant, such that higher symptom severity was associated with lower segmentation agreement, *B* = −0.001, *p* = 0.007; however, the fixed effect of Narrative type was not significant, *B* = 0.01, *p* = 0.093. Finally, the Symptom Severity × Narrative type interaction was not significant, *B* = −0.001, *p* = 0.06.

### Free recall

**Fig. 2:**
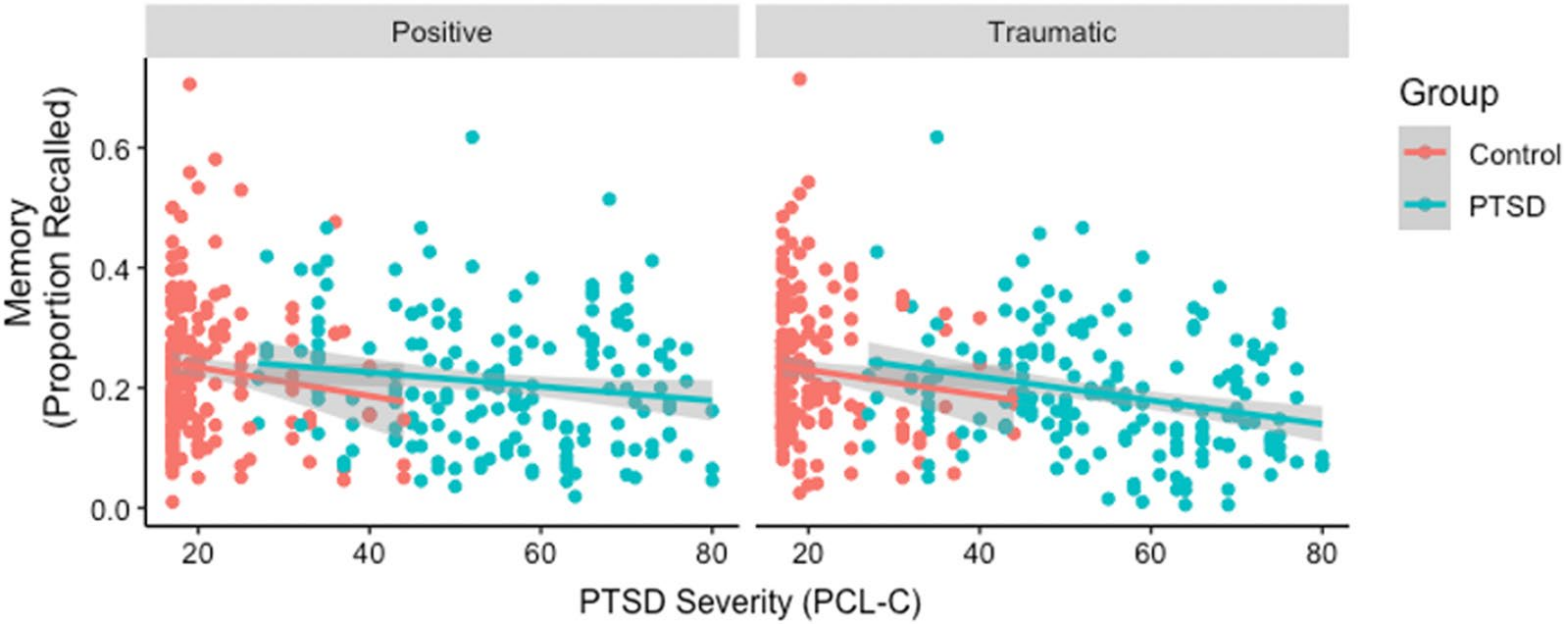
PTSD symptom severity was associated with lower memory performance after both traumatic and positive narrative priming. Blue dots = PTSD Group; Red dots = Control Group

Figure 2 plots the relationship between PTSD symptom severity and recall by group.

#### Group analysis

To evaluate the effects of PTSD and narrative priming on memory, we ran a mixed-effects model predicting the fixed effects of Group (PTSD vs. Control), Narrative Type (Traumatic vs. Positive), and their interaction on Memory. Subject and Video were treated as random effects. There was a significant effect of Group, such that those with PTSD (*M* = 0.20) recalled fewer actions than Controls (*M* = 0.23), *B* = −0.03, *p* = 0.037, but not of Narrative Type, *B* = −0.01, *p* = 0.095. The interaction of Group × Narrative type was not significant, *B* = −0.01, *p* = 0.477.

#### Symptom severity analysis

To evaluate the effects of PTSD symptom severity and narrative priming on memory, we ran a mixed-effects model predicting the fixed effects of Symptom Severity (mean-centered), Narrative Type (Traumatic vs. Positive), and their interaction on Memory. Subject and Video were treated as random effects. The effect of Symptom Severity was significant, such that higher symptom severity was associated with lower memory performance, *B* = −0.001, *p* = 0.004, as was the effect of Narrative type, *B* = −0.014, *p* = 0.002, however the interaction of Symptom Severity × Narrative Type, *B* = −0.000, *p* = 0.19, was not significant in predicting Memory.

### Mediation by segmentation

**Fig. 3:**
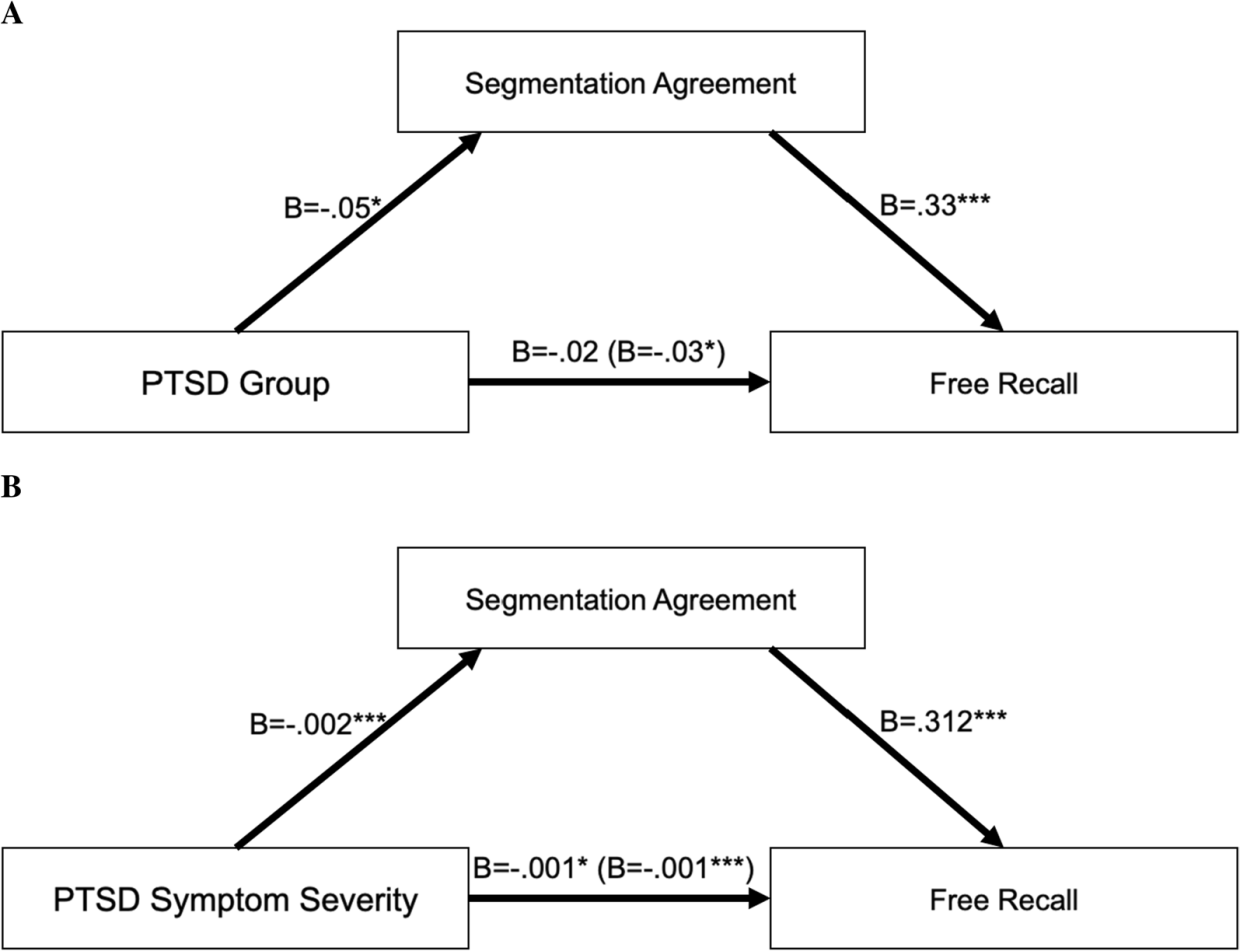
Segmentation agreement mediates the relationship between PTSD Group (**A**) or Symptom Severity (**B**) and Free Recall. Total effects are shown in parentheses. *p < .05; ***p < .001

In the final set of analyses, we tested whether segmentation mediated the relationship between PTSD and memory. Figure 3 shows the results of mediation models examining the direct and indirect effects of PTSD on Memory. Given that both Group and Symptom Severity significantly predicted memory in the section above, we conducted two sets of mediation analyses with either Group or Symptom Severity as the predictor. The *mediation* package in R (Tingley et al., 2014) allows one random effect per model. Therefore, in the following mediation analyses, we aggregated across video, such that each subject has one segmentation score (average segmentation ability across videos) and one recall score (average memory performance across videos).

#### Group analysis

We first evaluated whether segmentation agreement mediated the relationship between PTSD Group and Memory performance. Overall, Group and Segmentation agreement explained 27% of the variance in Memory. Group (PTSD vs. Control) explained a significant proportion of the variance in Memory performance, *B* = −0.03 (95% CI: −0.06, −0.01), *p* = 0.012, which can be decomposed into a significant indirect effect through Segmentation, *B* = −0.01 (95% CI: −0.03, 0.00), *p* = 0.036, and a non-significant direct effect, *B* = −0.02 (95% CI: -0.04, 0.00), *p* = 0.104. The mediator, Segmentation agreement, accounted for 45% of the total effect of Group on Memory. Therefore, the PTSD diagnosis was associated with lower segmentation agreement, which in turn was associated with lower memory performance. See Fig. 3a.

#### Symptom severity analysis

In the second mediation model, PTSD Symptom Severity and Segmentation Agreement explained 28% of the variance in Memory. Symptom Severity explained a significant proportion of the variance in Memory performance, *B* = −0.001 (95% CI: −0.002, −0.00), *p* < 0.001, which can be decomposed into a significant indirect effect through Segmentation *B* = −0.001 (95% CI: −0.001, 0.00), *p* < 0.001, and a significant direct effect, *B* = −0.001 (95% CI: −0.001, 0.00), *p* = 0.024. The mediator, Segmentation Agreement, accounted for 46% of the effect of Symptom Severity on Memory. Therefore, higher PTSD symptom severity was associated with lower segmentation agreement, which in turn was associated with lower memory performance. See Fig. 3b.

## Discussion

The current study investigated differences in event segmentation and memory for everyday activities in patients diagnosed with PTSD and people matched on age, gender, years of education, ethnicity, and trauma experience. We predicted that PTSD, as a diagnosis and as a continuous measure of symptom severity, would be related to poor event segmentation ability and long-term memory. We found that people diagnosed with PTSD had significantly worse memory than controls, and that across all participants higher PTSD symptom severity was associated with worse memory and worse segmentation. These symptom findings were consistent with Eisenberg et al. (2016), who found that PTSD symptom severity was associated with lower segmentation agreement and memory performance in a nonclinical sample. Our findings extend these results to a clinical sample with much higher symptom severity and show that symptom severity is more strongly associated with event cognition than the diagnosis itself.

Further, we predicted that these PTSD-related deficits in segmentation agreement and memory would be exacerbated after listening to a personal traumatic narrative relative to a personal positive narrative. We found that the priming manipulation did not affect segmentation agreement or memory, nor did priming interact with PTSD diagnosis or symptom severity. This is somewhat surprising given previous findings that listening to a script of one’s traumatic event increases arousal, as measured by skin conductance, and decreases activity in brain areas important for event cognition, such as the medial prefrontal cortex (Britton et al., 2005). We did observe that self-reported state-anxiety was higher after listening to the traumatic narrative priming than the positive narrative priming; however, participants’ increased anxiety did not affect their cognitive performance.

There are two viable explanations for this finding: (1) increased state anxiety does not affect event processing and immediate recall for everyday events; or (2) the narrative manipulation was not strong enough to produce effects on our dependent measures. Previous studies suggest that reminders of one’s trauma using video or auditory paradigms transiently increases arousal, stress response, and symptoms of PTSD, particularly arousal and re-experiencing symptoms (McNally et al., 1994; Rauch et al., 1996). Previous studies have also shown that these paradigms affect memory retrieval, at least for autobiographical information (McNally et al., 1994). Despite these findings, it does not appear that the increase in anxiety symptoms produced by the narrative priming task used in this study was enough to exacerbate the PTSD-related effects on event segmentation and memory. The cognitive effects and limits of this symptom provocation task warrant further study.

Our lack of traumatic priming effect is consistent with Eisenberg et al. (in prep) who found that priming individuals with their trauma narrative did not impact a person’s ability to predict what will come next in an everyday activity; an important ability in event processing. However, priming did produce significant changes in predictive eye movements, a covert eye-tracking measure of the extent to which participants look at an object immediately before the actor interacts with it. Thus, the priming manipulation appears to affect changes in attention to the unfolding situation, but it does not appear to affect behavioral measures of event processing. These results provide preliminary evidence that PTSD-related deficits in event-processing and memory reflect persistent cognitive deficits and are not transiently affected by heightened state anxiety; however, further research is needed to determine whether the manipulation was strong enough to elicit the hypothesized cognitive deficits.

Finally, as we predicted, segmentation agreement mediated the PTSD-related deficit in everyday memory performance. Both the diagnosis of PTSD and higher symptom severity was associated with lower segmentation agreement, which in turn was associated with lower memory performance. This suggests that deficits in event processing and encoding explain PTSD-related differences in memory for everyday events.

We know from the symptoms that define PTSD in the DSM 5 (APA, 2013) and theories that seek to explain PTSD (Brewin, 2011; Ehlers & Clark, 2000; Rubin et al., 2008; van der Kolk, 2007) that memory deficits are a hallmark symptom for people with PTSD, yet little research has investigated why this is the case. The results of our mediation analysis provide an initial answer; that trouble segmenting ongoing activity into discrete events is a major factor disrupting memory in PTSD. While we have reviewed several lines of research supporting the hypothesis that encoding deficits, such as event segmentation, are the cause of downstream memory problems associated with PTSD, there is also evidence of problems at consolidation (Kida, 2019) and retrieval (Wingenfeld et al., 2012). However, given the finding that event segmentation fully explains this relationship, deficits in consolidation and retrieval are likely inconsequential after differences in the encoding mechanism, event segmentation, are accounted for.

Of note, Sherrill et al. (2019) found that segmentation agreement mediated the relationship between state anxiety and memory, such that heightened state anxiety from viewing a stressful video increased segmentation agreement, which in turn decreased memory performance. Further exploratory analyses revealed that heightened state anxiety enhanced attention to perceptual changes over conceptual changes. This disparate finding indicates that while event segmentation is an important process to consider when evaluating memory, state anxiety and PTSD seems to affect event processing in different ways. It may be that acute stress, as instigated by the stressful event segmentation paradigm (Sherrill et al., 2019) improves event segmentation, while chronic stress from PTSD impairs event segmentation. More research is needed to better understand these relationships.

An important contribution of the current research is that it documents effects of PTSD diagnosis and symptom severity on memory for everyday activity that is unrelated to a person’s traumatic experiences. The modest number of previous studies examining effects of PTSD on memory for non-trauma-related material have reported small to moderate PTSD-related deficits in memory measures, with considerable variability (Brewin et al., 2007). Our findings support the presence of a pervasive deficit in event processing and memory that affects how people with PTSD perceive and remember everyday, non-emotional events. The mechanism of this deficit remains to be explored, but recent findings from Eisenberg et al. (in prep) suggest that people with PTSD may fail to adequately monitor prediction error (i.e., when ongoing activity does not match predicted activity), which would render segmentation less reliable. This is supported by their finding that both overt predictions of what will come next and covert predictive looking are altered in people with PTSD. According to Event Segmentation Theory (Zacks et al., 2007), this inability to predict what will happen next may make people with PTSD unable to efficiently segment ongoing activity into meaningful chunks of activity, which would affect moment-to-moment comprehension and long-term memory of the activity. These deficits in event processing and memory may explain subjective memory complaints for everyday information in people with PTSD (Carlozzi et al., 2011).

While the present findings are specific to non-traumatic, everyday memory, they suggest a general episodic memory deficit that would also impair how traumatic events are encoded and remembered. It is unclear how event processing is changed by arousal during a traumatic event. However, work by Sherrill et al. (2019) suggests that heightened arousal changes the relative ratio of perceptual and conceptual processing, which has downstream effects on event segmentation and memory. Therefore, event segmentation and memory for traumatic events are likely to be particularly worse for people with PTSD given the general processing deficits we show here with everyday stimuli.

Further, our findings highlight the limitations of categorical diagnoses. While we did find differences in event processing and memory abilities based on diagnosis, symptom severity proved to be a better predictor of these relationships. Previous studies have also found PTSD symptom severity to be an important predictor of occupational functioning following accidental injuries in clinical and nonclinical groups (Mathews & Chinnery, 2005). In fact, individuals with subclinical PTSD reported lower functioning than those with no PTSD, suggesting that this intermediate group may also be at risk of poor outcomes. These findings highlight the variability in symptom presentation within categorical diagnoses and the implications of this variability on functional outcomes.

Previous studies suggest that reminders of one’s trauma using video or auditory paradigms transiently increases arousal, stress response, and symptoms of PTSD, particularly arousal and re-experiencing symptoms (McNally et al., 1994; Rauch et al., 1996). Previous studies have also shown that these paradigms affect memory retrieval, at least for autobiographical information (McNally et al., 1994). Despite these findings, it does not appear that the increase in anxiety symptoms produced by the narrative priming task used in this study was enough to exacerbate the PTSD-related effects on event segmentation and memory. The cognitive effects and limits of this symptom provocation task warrant further study.

There are some limitations to our findings. First, PTSD diagnoses were based on a version of the SCID-IV that was modified to match symptoms that appear in the DSM-5. Although we believe the wording used here was able to elicit similar responses to those elicited by the SCID-5 (which was not available at the time this study was begun), it is unclear how these responses and diagnoses would actually align. Second, the narrative priming was given to participants eleven times during each session, which may have resulted in habituation to the traumatic narrative. We do not feel that this was a problem as anxiety ratings did not decline with multiple presentations; however, a more objective measure of arousal that does not rely on self-report, such as skin conductance, may be a better way to measure the effect of repeated presentation of the narratives. Third, differences in segmentation ability and memory performance may be due to PTSD-related difficulties with attention and concentration, as these are symptoms of PTSD. If people with PTSD are unable to attend to the important details of a scene, they might miss important cues about activity changes, which would affect event segmentation, and miss information about the scene as a whole, which would affect memory for the scene. Since we do not have concurrent measures of task-related attention and concentration, we cannot say unequivocally that performance on our cognitive measures were not primarily due to deficits in these areas. However, the eye-tracking apparatus used during the experiment required that participants maintain their gaze in the direction of the screen and therefore made complete inattention to the task improbable. Lastly, we did not collect data on income, education, or socioeconomic status. These may be an important variables to consider in the future as they have been found to be related to both cognitive abilities (Leonard et al., 2015) and PTSD (DiGrande et al., 2008).

To our knowledge, this is the first study to investigate PTSD-related deficits in event segmentation or memory for everyday events using ecologically valid stimuli. We found that higher PTSD symptom severity was associated with worse segmentation agreement and memory for everyday events portrayed in videos. Our findings support the idea that people with PTSD have trouble remembering non-emotional events (Eisenberg et al., 2016) and indicate that there are discernable impairments in both encoding and retrieval. It is notable that we found these deficits in mundane everyday stimuli that depict real-world events. Thus, having a diagnosis of PTSD and/or more severe PTSD symptoms can impede one’s ability to encode and remember everyday information, which can strongly impair one’s ability to function.

Deficits in event encoding and retrieval are clinically meaningful because they are associated with both PTSD diagnosis and symptom severity. Based on these findings, developing an event segmentation intervention may be an effective way to treat cognitive deficits associated with PTSD (see also, Sherrill & Magliano, 2017). For example, Gold et al. (2017) found that cueing event boundaries improved memory in older adults, which is another population with poor segmentation ability. Further research is needed to determine whether segmentation training would alleviate these cognitive deficits in those who experience PTSD symptoms. Finally, Sherrill and Magliano (2017) claimed that established treatments for PTSD, such as prolonged exposure therapy and cognitive processing therapy, that improve organization of trauma memories are likely leveraging segmentation processes. Our findings suggest that event segmentation principles may provide some insights into improving such treatments and extend them to non-traumatic, everyday experiences.

## Open practices statements

The data and materials for all experiments reported in this manuscript are available at https://osf.io/ht4fg/. None of the experiments were preregistered.

## Supplementary Information

**Additional file 1**. **Supplemental Figure 1.** Higher PTSD Symptom severity was associated with higher state-anxiety, especially after the Traumatic narrative. Blue = PTSD Group; Red = Control Group.

## Data Availability

The data and materials for all experiments reported in this manuscript are available at https://osf.io/ht4fg/. None of the experiments were preregistered.
