# Supplementary material for: PTSD is associated with impaired event processing and memory for everyday events
Source: Cogn Res Princ Implic. 2022 Apr 25;7:35. doi: 10.1186/s41235-022-00386-6 (PMC9038970; doi:10.1186/s41235-022-00386-6)
Supplement: Supplementary file 1 — Additional file 1. Supplemental Figure 1. Higher PTSD Symptom severity was associated with higher state-anxiety, especially after the Traumatic narrative. Blue = PTSD Group; Red = Control Group. [file 41235_2022_386_MOESM1_ESM.docx]

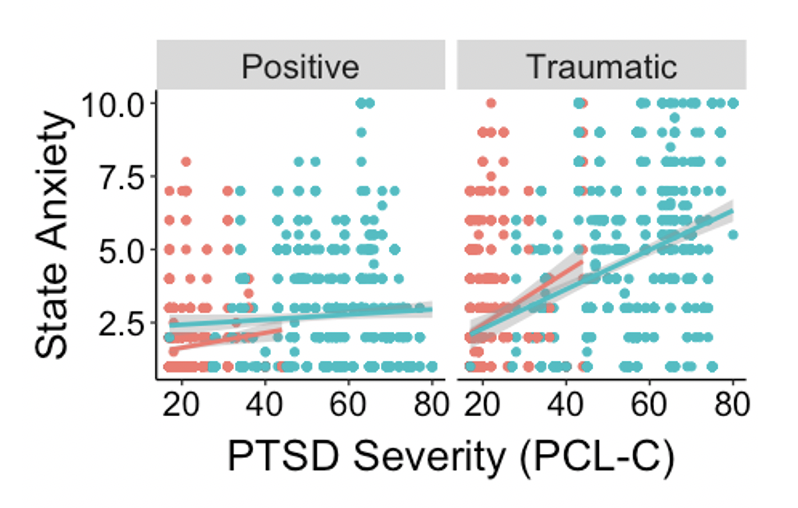


Supplemental Figure 1. Higher PTSD Symptom severity was associated with higher state-anxiety, especially after the Traumatic narrative. Blue = PTSD Group; Red = Control Group
